# Smoking bans in mental health hospitals in Japan: barriers to implementation

**DOI:** 10.1186/s12991-015-0076-9

**Published:** 2015-10-29

**Authors:** Kazumichi Hashimoto, Manabu Makinodan, Yasuhiro Matsuda, Tsubasa Morimoto, Shotaro Ueda, Toshifumi Kishimoto

**Affiliations:** Department of Psychiatry, Faculty of Medicine, Nara Medical University, 840 Shijocho Kashihara, Nara, 634-8522 Japan

**Keywords:** Smoking ban, Mental health hospital, Psychiatric symptoms, Tobacco, Japan

## Abstract

**Background:**

A number of studies have reported that smoking rates are higher and smoking cessation rates are lower in patients with mental disorders than in the general population. Despite the harmful effects of smoking, implementing total smoking bans in mental health hospitals is difficult. We investigate the status of smoking bans and the barriers to the implementation of total smoking bans in Japanese mental health hospitals.

**Methods:**

A questionnaire survey was administered to the directors of 1242 Japanese mental health hospitals in March 2013.

**Results:**

Forty-nine percent (*n* = 612) of the hospital directors responded. Of these, 24 % implemented total smoking bans and 14 % limited the bans to hospital buildings. In 66 and 68 % of the remaining hospitals, smoking rooms were located in open and closed wards, respectively, and completely separate from nonsmoking areas. Hospitals that had not implemented total smoking bans were concerned that introducing a total ban would exacerbate patients’ psychiatric symptoms (46 %) or increase the incidence of surreptitious smoking (65 %). However, of the hospitals that had implemented total smoking bans, only 2 and 30 % identified “aggravation of psychiatric symptoms” and “increased surreptitious smoking” as disadvantages, respectively. The other concerns regarding the implementation of total smoking bans were staff opposition (21 %) and incidence of smoking around hospital grounds (46 %). These concerns were overcome by educating staff about smoking and cleaning the area around the hospital.

**Conclusions:**

There are some barriers to implementing total smoking bans in Japanese mental health hospitals. However, our study indicates that implementation of total smoking bans in mental health hospitals was minimally problematic and that barriers to the implementation of smoking bans could be overcome. As the current number of hospitals that have implemented total smoking bans is low in Japan, more hospitals should introduce total smoking bans.

**Electronic supplementary material:**

The online version of this article (doi:10.1186/s12991-015-0076-9) contains supplementary material, which is available to authorized users.

## Background

Cigarette smoking is the leading cause of preventable death and a considerable public health problem worldwide [1]. The World Health Organization Framework Convention on Tobacco Control came into force in 2005 to protect present and future generations from the devastating health, social, environmental, and economic consequences of tobacco use and exposure to tobacco smoke [2]. Recently, the provision of legislation to facilitate smoke-free policies in the workplace and public places has increased in a number of countries [3]. In Japan, the Health Promotion Act was introduced in 2002 to maintain health and prevent the spread of modern disease [4]. According to this law, superintendents of facilities used by a large number of people—such as schools, hospitals, and public amenities—are required to protect users from secondhand smoke, which is a risk factor for lung cancer [5] and heart disease mortality [6]. In addition, the notice “Concerning Measures for Passive Smoking Prevention” was issued by the Director General of the Health Service Bureau (2010), and municipalities established regulations to introduce a total smoking ban in public places with heavy human traffic to reduce passive smoking. However, compliance with these laws was not obligatory.

Previous studies have reported that smoking rates are higher and smoking cessation rates are lower in people with mental disorders than in mentally healthy individuals [7, 8]. Consequently, the former have a high risk of death due to smoking. Individuals with major depression, alcohol disorders, and schizophrenia have been found to have high mortality rates for vascular diseases and cancer [9]. However, for many decades in mental health hospitals in Japan, cigarette smoking has been permitted as a largely acceptable practice and has been considered a matter of personal preference [10]. This may be related to the unique position occupied by smoking within the practice and culture of psychiatric care [11]. Further, there are barriers to implementing total smoking bans in mental health settings. They are concerns such as the anticipated increase in aggression and psychiatric symptoms, opposition of staff who smoke [12], and the belief that clandestine smoking constitutes an enhanced fire hazard risk [3].

In two prefectures in Japan, legislation imposing smoking bans is enforced in public spaces. In one of them, a smoking room completely separated from nonsmoking areas in all hospitals is allowed. In the other, smoking is allowed in psychiatric units. The Japan Council for Quality Health Care also compelled general hospitals to introduce total smoking bans except for psychiatric units. Thus, Japanese mental health hospitals are clearly exempt from these regulations. Although smoking bans are poorly implemented in Japanese mental health hospitals, no study has examined the status of smoking bans in these care settings.

In America, the Mayo Medical Center, including its psychiatric units, implemented a smoke-free policy in 1989 [13]. In Switzerland, smoking rooms were removed and smoking was completely prohibited indoors in psychiatric units in 2006 [14]. However, in Japan, the first total smoking ban in a mental health hospital was implemented only in 2004 [10]. The implementation of smoke-free policies in mental health hospitals began considerably later in Japan than other countries.

While both people with and without mental disorders should be protected from smoking-related harm, the implementation of total smoking bans in mental health hospitals in Japan seems to be suboptimal relative to general hospital settings. This suggests that there are unique attitudinal barriers to the implementation of total smoking bans within mental health settings in Japan, as in other countries [15]. Accordingly, the present study examined the status of smoking bans in mental health hospitals and assessed attitudes toward total smoking bans in Japan. We defined the term “smoking ban” to mean the prohibition of smoking regardless of the presence or absence of penalty.

## Methods

### Design and setting

A cross-sectional study was conducted. A list of private mental health hospitals in Japan, which included 1208 institutions, was obtained from the Japanese Association of Psychiatric Hospitals, and a list of public 34 mental health hospitals composed primarily of psychiatric units was obtained from the Japan Municipal Hospital Association website (https://www.jmha.or.jp/jmha/). A total of 1242 mental health hospitals were included in this study. Some had long-term care wards and/or general wards other than psychiatric wards.

### Procedures

Questionnaires were mailed to the hospital directors at the beginning of March 2013, and we asked them to complete the survey and return the responses by March 31, 2013.

### Measures

The questionnaire was designed to collect information on the attitude to smoking bans in mental health hospitals, referring to previous research on this topic [7, 16]. Questionnaires asked about the following: (1) the status of smoking bans in hospitals in March 2013, (2) plans to introduce a smoking ban in the future, (3) reasons for the hospital’s reluctance to implement a total smoking ban, (4) the support provided to hospital patients to facilitate smoking cessation, (5) the hospital’s reason for introducing a total smoking ban, (6) perceived advantages of introducing a total smoking ban, and (7) perceived disadvantages of introducing a total smoking ban. The questionnaire consisted of multiple-choice questions, and if none of the choices corresponded with participants’ answers, they could choose “other” and enter their own responses. Questions 1 and 4 were applicable to all hospitals; Questions 2 and 3 were applicable to hospitals that had not implemented total smoking bans; Questions 5, 6, and 7 were applicable to the hospitals that had implemented total smoking bans.

The primary outcome measure was the proportion of the mental health hospitals that had implemented total smoking bans. The secondary outcome measure was the attitude to smoking bans in mental health hospitals reported via the questionnaire.

### Data analysis

Questionnaire responses were coded and entered into SPSS (v. 18). We compared the mean number of inpatient beds and distribution of hospital location between respondents and non-respondents, using a *t* test and a Chi-square test, respectively.

## Results

### Smoking ban status in mental health hospitals

**Fig. 1:**
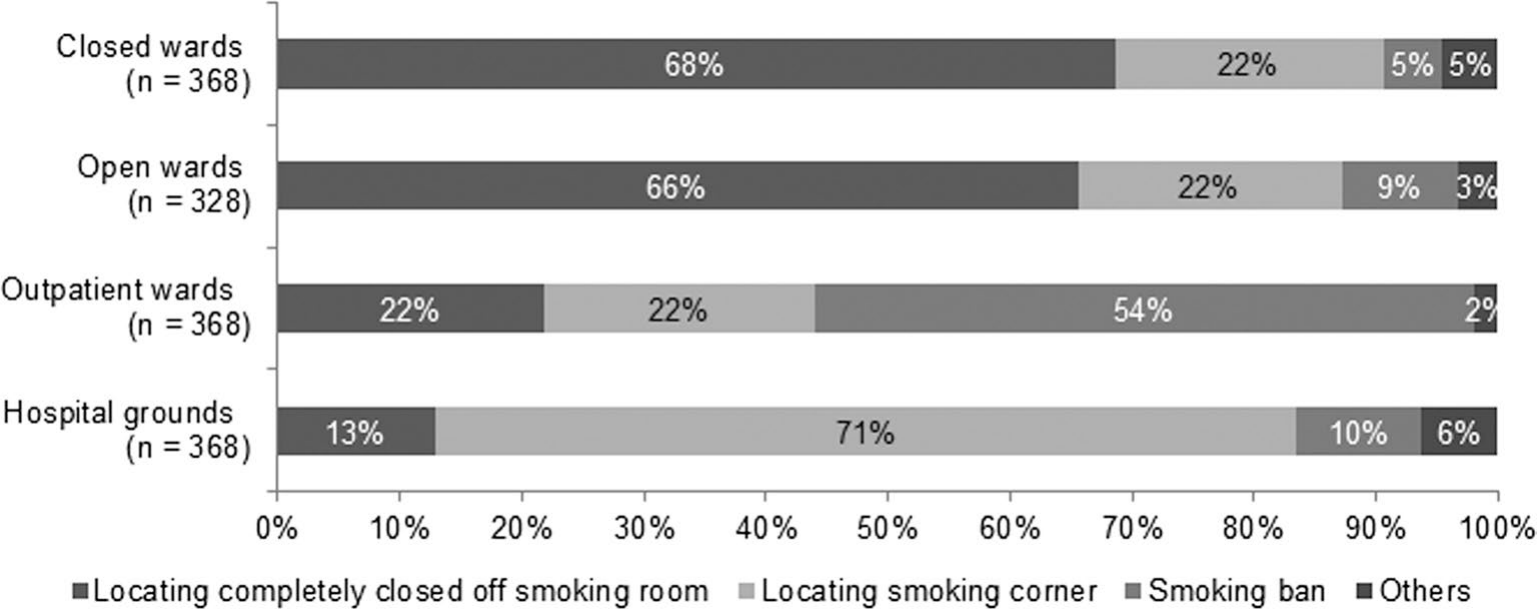
Smoking arrangements in hospitals where smoking bans do not encompass the entire grounds or where bans are limited to hospital buildings

**Fig. 2:**
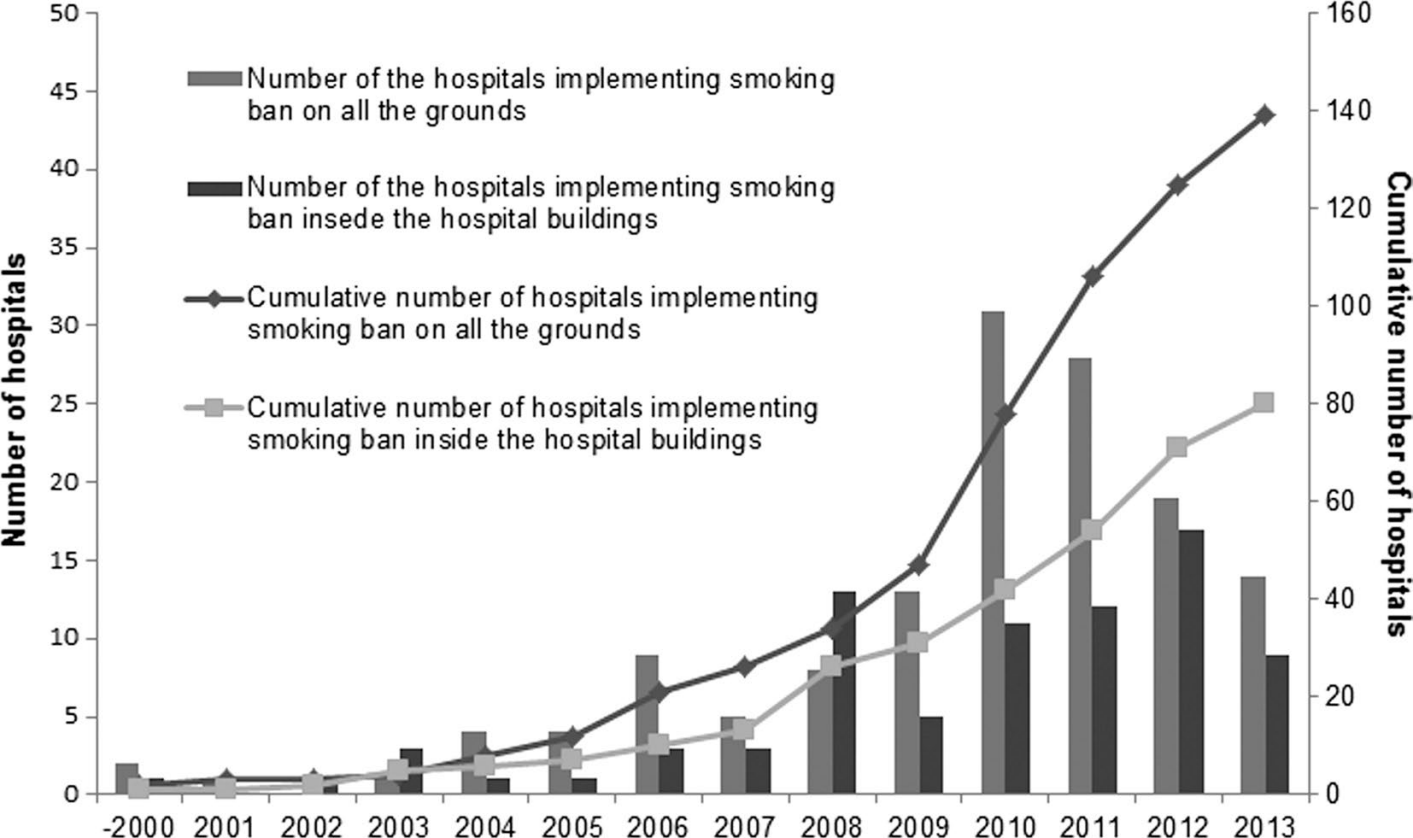
Change in the number of the hospitals implementing smoking bans

The response rate was 49.3 % (612 out of 1242 hospitals). The number of inpatient beds in the participating hospitals is summarized in Additional file 1: Table S1. As of March 2013, 23.5 % of the participating hospitals had implemented total smoking bans, and 14.4 % had limited the implementation of smoking bans to hospital buildings. Implementation of smoking bans did not significantly differ between public and private hospitals (*χ*^2^ = 0.53, *p* = 0.75). Of the hospitals that had not implemented total smoking bans, 7.7 % planned to do so in the future. Figure 1 shows the smoking arrangements established by hospitals that had not implemented smoking bans encompassing their entire grounds or those with bans limited to hospital buildings. In 68 and 66 % of these hospitals, smoking rooms were located in closed and open wards, respectively, and were completely separate from nonsmoking areas. In addition, 54 % reported that smoking bans were in force in their outpatient wards, and 71 % reported that smoking areas were located outside hospital buildings, close to outdoor nonsmoking areas in the hospital grounds. Further, 2.9 % of all participating hospitals had simply established smoking rooms next to nonsmoking areas. Approximately, 75 % of the hospitals that had introduced total smoking bans had done so after 2010 (Fig. 2).

### Questionnaire concerning the implementation of smoking bans

Hospitals that had not implemented total smoking bans were concerned that introducing a ban would exacerbate patients’ psychiatric symptoms (45.1 %) or increase the incidence of surreptitious smoking (65.0 %; Additional file 1: Table S2). However, only 1.7 and 30.2 % of hospitals that had implemented total smoking bans cited “aggravation of psychiatric symptoms” and “increased surreptitious smoking” as disadvantages (Additional file 1: Table S3). Another concern regarding the implementation of smoking bans was that the incidence of smoking around hospital grounds would increase (46.0 %). In addition, the main disadvantage of total smoking bans (52.6 %), cited by the hospitals that had implemented them, was that residents living nearby complained about people smoking outside hospital grounds, near their homes. In addition, 59.4 and 20.9 % of the hospitals that had not implemented total smoking bans expressed concern that inpatients and staff members, respectively, would not consent to a ban. Some respondents reported that total smoking bans should not be enforced, as they violate patients’ rights to smoke. Approximately, 70 % of the hospitals provided smoking cessation support for patients (Additional file 1: Table S4); they also provided nicotine patches at low prices and held smoking cessation workshops for staff members.

The hospitals’ reasons for implementing total smoking bans are described in Additional file 1: Table S5. Almost 90 % of the hospitals that had implemented total smoking bans identified the social trend toward smoking bans in the healthcare field as the reason for the implementation of total bans. In addition, 26.8 % reported that they had implemented a total smoking ban following an evaluation by the Japan Council for Quality Health Care. Other reasons included compliance with local regulations or national notices and prevention of problems caused by smoking, such as fire and stained hospital walls.

The perceived advantages of implementing a total smoking ban are shown in Additional file 1: Table S6. A total of 75.2 % of respondents reported that the cleanliness of their hospitals had improved, 56 % reported a reduction in staff smoking rates, and 53.9 % reported a decrease in problems caused by smoking. Importantly, a few respondents reported that they had received positive feedback regarding the ban from patients (14.9 %) and residents living nearby (4.3 %). Other advantages included reduced costs and effort over cigarettes, a decrease in secondhand smoke, and health benefits.

The disadvantages of total smoking bans are described in Additional file 1: Table S3; 44.8 % of respondents chose “other,” while approximately 30 % reported that they had not experienced any problems related to the implementation of a total smoking ban.

## Discussion

This study investigated the status of smoking bans in Japanese mental health hospitals. A total of 23.5 % of the participating mental health hospitals had implemented total smoking bans when the survey was conducted in March 2013, and the number of mental health hospitals that had implemented total smoking bans had increased since 2010. This increase could have been related to the notice “Concerning Measures for Passive Smoking Prevention” issued by the Director General of the Health Service Bureau on February 25, 2010 [17]. However, compliance with this notice was voluntary. Yamato et al. reported that 86 % of Japanese university hospitals with psychiatric wards had implemented total smoking bans in 2011. In the United Kingdom, 13 % of mental health establishments and 41 % of acute trusts have implemented smoke-free policies that apply to the entire premises, including the hospital grounds, without exemption [3]. In the United States, 88.1 % of psychiatric/alcohol-chemical dependency hospitals and 95 % of general hospitals complied with smoking ban standards [18]. As the differences in the prevalence of smoking bans between general and mental health hospitals varied between countries, implementation of smoking bans appears to be more difficult in psychiatric than general hospitals. A number of studies have reported obstacles to the implementation of total smoking bans in mental health hospitals [15, 19].

The present results indicated that directors of Japanese hospitals were concerned that an introduction of a total smoking ban would exacerbate patients’ psychiatric symptoms; however, only a small number of directors from the hospitals that had implemented total smoking bans reported this outcome. Similarly, Wye et al. reported that in Australian mental health hospitals, the greatest barriers to successful total smoking ban implementation were fear of aggression and noncompliance in patients [20]. Cole et al. conducted their research at a New York state psychiatric facility and demonstrated a small but statistically significant decrease in global assessment of function scores but no significant changes in baseline Brief Psychiatric Rating Scale scores, after the implementation of a smoke-free policy [21]. A discrepancy between anticipated and observed exacerbation of psychiatric symptoms has also been reported [19, 22, 23].

Our results were congruent with those of other studies indicating concern regarding staff opposition to implementing a total smoking ban [20, 24]. This may be partly due to a lack of knowledge among or higher incidence of smoking in mental health hospital staff members than those of other hospitals [19]. A number of previous studies reported that staff members who smoked were more likely to hold negative beliefs regarding smoke-free policies [11, 25]. Staff acceptance of total smoking bans has been associated with successful implementation of smoking bans [26]. Therefore, some studies have noted that training staff members to recognize and manage nicotine withdrawal and relapse, and supporting individuals in smoking cessation are important factors in successful implementation of smoking bans [11, 20, 26]. In our study, 20.4 % of respondents reported staff opposition to be a barrier to implementing smoking bans; it may be important to educate them to increase the implementation of total smoking bans in Japanese mental health hospitals.

In accordance with previous research findings [20], 75 % of respondents reported that the cleanliness of their hospitals improved following the implementation of total smoking bans. Approximately, 50 % of the respondents from hospitals that had implemented total smoking bans reported that they had received complaints from residents living nearby because of an increase in smoking outside hospital grounds, near their homes. Regarding the advantages of total smoking bans, a few respondents chose “receiving positive feedback from residents living nearby.” To address this complaint, some hospitals reported that staff cleaned the areas outside hospital grounds regularly. While a total smoking ban was previously reported to reduce staff members’ workload [20], respondents in this study reported that the implementation of total smoking bans created additional work for staff members, such as cleaning areas outside hospital grounds because of an increase in smoking in these areas.

Secondhand smoke exposure, like firsthand smoke, causes death, diseases, and disability [27] and only total smoking bans can protect people from secondhand smoke [28]. At this point, in mental health settings, patients in closed wards are exposed to secondhand smoke. We consider it important for all patients, especially in mental health settings, for total smoking bans to be implemented. As mentioned above, our research indicated some obstacles that Japanese directors had concerns about. Previous studies reported that the barriers to implementing smoking bans were issues related to patients’ rights and opposition from staff who smoke and who believe that a smoke-free policy would adversely affect psychiatric patients’ mental health [15]. The key features in mental health hospitals where smoking bans have been effectively implemented are staff education and support, patient preparation, provision of nicotine replacement therapy (NRT), establishment of overarching strategy aimed at long-term quitting with evidence, and changes in smoking culture [26, 29–31]. However, NRT is covered by health insurance for outpatients but not for inpatients in all hospitals, including pubic hospitals. Additionally, in the Japanese mental health hospitals that successfully implemented total smoking bans, the strong leadership of committees, including the directors, has overcome the barriers to smoking bans by educating patients and staff in workgroups [10]. Among the developed countries (e.g., G8), only Japan has no national law imposing smoking bans in public places [32]. To improve the implementation of total smoking bans in mental health hospitals, the establishment of a national law that obligates smoking bans may be necessary.

This study had some limitations. First, approximately 50 % of the potential participants did not return the questionnaire. There were no significant differences in the location of hospitals (*χ*^2^ = 4.21, *p* = 0.52) and the number of beds (*t* = 1.14, *p* = 0.26) between responding and non-responding hospitals. However, the hospitals that did not answer the questionnaire were not likely to have implemented smoking bans; therefore, we may have overestimated the proportion of mental health hospitals that had implemented smoking bans. Further, we sent no reminders to the directors of hospitals, so the response rate could be improved by sending reminders. Second, the questionnaires were sent to hospital directors, and their beliefs may differ from those of clinical staff working in mental health hospitals. Third, the questions about the implementation of total smoking bans differed between the hospitals that implemented total smoking bans and those that did not. Therefore, we could not directly compare directors’ attitude toward total smoking bans between these mental health settings, and could not detect which hospitals are more likely to undertake total smoking bans.

## Conclusions

In this study, we shed light on the status of smoking bans in Japanese mental health hospitals and revealed their directors’ attitudes to implementing total smoking bans. The results indicated that concerns regarding the introduction of total smoking bans were less problematic than expected in hospitals that had already implemented bans. As the number of hospitals that have implemented total smoking bans is low in Japan, our findings show the need for increased implementation of total smoking bans in mental health hospitals.

## Additional file

10.1186/s12991-015-0076-9 The respondent characteristics (**Table S1**) and the responses to the questionnaire on the implementation of smoking bans (**Tables S2–S6**).
