# Supplementary material for: Smoking bans in mental health hospitals in Japan: barriers to implementation
Source: Ann Gen Psychiatry. 2015 Oct 29;14:35. doi: 10.1186/s12991-015-0076-9 (PMC4625877; doi:10.1186/s12991-015-0076-9)
Supplement: Supplementary file 1 — 10.1186/s12991-015-0076-9 The respondent characteristics (Table S1) and the responses to the questionnaire on the implementation of smoking bans (Tables S2–S6). [file 12991_2015_76_MOESM1_ESM.docx]

Table S1. Respondent characteristics

| **Survey items** | **Number of respondents (%)** |
| --- | --- |
| **Number of inpatient beds** |  |
| **≤**99 beds | 24 (3.9) |
| 100–199 beds | 194 (31.9) |
| 200–299 beds | 211 (34.7) |
| 300–399 beds | 90 (14.8) |
| 400–499 beds | 57 (9.4) |
| ≥500 beds | 32 (5.3) |
| **Status of smoking ban** |  |
| All hospital grounds | 144 (23.5) |
| Inside the hospital buildings | 88 (14.4) |
| Others | 380 (62.1) |
| **Plan for the future*** |  |
| All hospital grounds | 47 (10.0) |
| Inside the hospital buildings | 38 (8.1) |
| No change | 345 (73.7) |
| Others | 24 (5.1) |
| Non-response | 14 (3.0) |

* Percentages refer to total number of respondents who had not implemented a smoking ban on all hospital grounds (n = 468).

Table S2. Reasons for not implementing a smoking ban on all hospital grounds*

| **Decision branches** | **(n = 446)** |
| --- | --- |
| Afraid to aggravate psychiatric symptoms | 45.1% |
| Afraid of people sneaking a smoke | 65.0% |
| Staff resistance | 20.9% |
| Patient resistance | 59.4% |
| Patients’ family resistance | 3.4% |
| Afraid of increased smoking around hospital grounds | 46.0% |
| Lack of knowledge about the way to accomplish a smoking ban on all hospital grounds | 4.9% |
| Afraid of a decrease in the number of admissions of smoking patients | 9.6% |
| Have never thought about a smoking ban on all hospital grounds | 10.1% |
| Others | 14.3% |

Table S2 includes the hospitals that had not implemented total smoking bans (n = 468)

* Each hospital could select multiple responses.

Table S3. Disadvantages of implementing a smoking ban on all hospital grounds*

| **Decision branches** | **(n = 116)** |
| --- | --- |
| Receiving a complaint from people living around the hospital due to increased smoking outside the hospital grounds | 52.6% |
| Increase of patients who sneak smokes in the hospital | 30.2% |
| There are some patients who desire a discharge or change of hospital | 6.9% |
| Aggravation of patients’ psychiatric symptoms because of smoking cessation | 1.7% |
| There is no end to the patients and staff members who cannot adhere to the smoking ban | 21.6% |
| Others | 44.8% |

Table S3 includes only hospitals that had implemented total smoking bans (n = 144).

* Each hospital could select multiple responses.

Table S4. Support provided to patients to quit tobacco smoking in hospitals*

| **Decision branches** | **(n = 587)** |
| --- | --- |
| Provision of information about smoking and health | 51.1% |
| Individualized instruction for outpatients | 21.3% |
| Individualized instruction for inpatients | 34.8% |
| Group instruction | 9.2% |
| Individualized instruction in smoking cessation outpatient service | 7.8% |
| Other support | 2.9% |
| No support | 29.8% |

Table S4 includes all hospitals (n = 612).

* Each hospital could select multiple responses.

Table S5. Reasons for deciding to implement a smoking ban on all hospital grounds*

| **Decision branches** | **(n = 142)** |
| --- | --- |
| With the rebuilding or new construction of hospital | 16.2% |
| With the evaluation for healthcare functions of the hospital by the Japan Council for Quality Health Care | 26.8% |
| For social trends about healthcare toward a smoking ban (e.g., prevention of secondhand smoking) | 88.0% |
| For the foundation of smoking cessation outpatient service | 16.2% |
| With the restructuring of hospital units (ex. new construction of emergency psychiatric unit) | 3.5% |
| Others | 16.2% |

Table S5 includes only hospitals that had implemented total smoking bans (n = 144).

* Each hospital could select multiple responses.

Table S6. Advantages of smoking bans on all hospital grounds*

| **Decision branches** | **(n = 141)** |
| --- | --- |
| Decrease in trouble caused by cigarette smoking | 53.9% |
| Hospitals became clean | 75.2% |
| Received a good evaluation by patients’ families | 32.6% |
| Received a good evaluation by patients | 14.9% |
| Received a good evaluation by the people living around the hospital | 4.3% |
| Decrease in psychotropic drug dosage | 6.4% |
| Decrease in smoking rate of staff members | 56% |
| Others | 12% |

Table S6 includes only hospitals that had implemented total smoking bans (n = 144).

* Each hospital could select multiple responses.
